# Progesterone and Cilostazol Protect Mice Pancreatic Islets from Oxidative Stress Induced by Hydrogen Peroxide 

**Published:** 2014

**Authors:** Akram Ahangarpour, Hamid Heidari, Seyyed Ali Mard, Mahmoud Hashemitabar, Ali Khodadadi

**Affiliations:** a *Health Research Institute, Diabetes Research Center, Department of Physiology, Ahvaz Jundishapur University of Medical Sciences, Ahvaz, Iran. *; b *Cellular and Molecular Research Center (CMRC), Department of Physiology, Faculty of Medical Sciences, Ahvaz Jundishapur University of Medical Sciences, Ahvaz; Iran. *; c *Physiology Research Center (PRC), Research Institute for Infectious Diseases of Digestive System and Department of Physiology, School of Medicine, Ahvaz Jundishapur University of Medical Sciences, Ahvaz, Iran. *; d *Cellular and Molecular Research Center (CMRC), Department of Anatomical Science, Ahvaz Jundishapur University of Medical Sciences, Faculty of Medicine, Ahvaz, Iran. *; e *Cellular and Molecular Research Center (CMRC), Department of Immunology Science, Ahvaz Jundishapur University of Medical Sciences, Faculty of Medicine, Ahvaz, Iran. *

**Keywords:** Cilostazol, H_2_O_2_, Insulin, Islet, Progesterone

## Abstract

Reactive oxygen species and oxidative stress impair β-cell function and reduce insulin secretion. It has been shown that progesterone and cilostazol possess antioxidant properties. The present study was aimed to investigate *in-vitro *pretreatment effect of progesterone and cilostazol on insulin secretion as well as their protective effects against hydrogen peroxide-induced oxidative stress in pancreatic isolated islets from mouse.

Pancreatic islets were isolated from 84 male NMRI mice (25-30 g) by collagenase digestion method and pretreated for 48 h with cilostazol (10 μM), progesterone (0.5 μM) and glibenclamide (10 μM) in culture medium. Then islets were exposed to hydrogen peroxide (H_2_O_2_. 500 μM) for 2 h. Next, culture mediums containing glucose concentration of 2.8 mM or 16.7 mM were added to them and incubated in this status for 1 h. At the end, the rate of insulin output from islets, lipid peroxidation and antioxidant enzymes activities in islet tissues were assayed.

Exposure of islets to H_2_O_2_, resulted in a significant decrease in insulin secretion, superoxide dismutase and catalase activities (P < 0.001). Also islets malondialdehyde levels were increased by H_2_O_2_, after addition of 2.8 mM (P < 0.05) and 16.7 mM (P < 0.001) glucose. 48 h pretreatment of islets with cilostazol and progesterone, significantly reverted back this changes (P < 0.05).

Results of present study showed that cilostazol and progesterone protect mice pancreatic islets against H_2_O_2_-induced oxidative stress. At the end, our results suggested that protective effects of progesterone and cilostazol are mediated by augmentation the antioxidant defence system of islets.

## Introduction

Diabetes is one of the serious endocrine disorders in worldwide and has predicted that its prevalence will increase noticeably by the year 2030 (1).

*In-vivo *studies have indicated that excessive generation of reactive oxygen species (ROS) occurs in diabetes situation. Also oxidative stress is accompanied by imbalance between oxidant and antioxidant systems, has a critical role in development of this disease and lead to destruction of insulin producing pancreatic β-cells (2, 3).

Since β-cells, contain very low level of antioxidant defense enzymes such as superoxide dismutase (SOD) and catalase (CAT), these cells have extremely great sensitivity to free radical induced damage. On the other hand, elevations of cell antioxidant enzyme activities lead to protection against ROS (4). Also one of the detrimental effects of ROS is lipid peroxidation that may result in β-cells death and loss of insulin secretion through apoptosis process (5). So it seems that exposure of pancreatic islets to exogenous insulinotropic and antioxidant agents is very important in treatment strategies for diabetes. Several studies have shown that progesterone (PRO) possesses antioxidant properties such as scavengering of ROS in cancer cells (6) and increasing SOD activity in human endometrial stromal cells (7). Also Morrissy and *et al. *reported that progesterone can induce antioxidant genes expression in cardiomyocytes cells and exert antioxidant and antiapoptic effects (8).

Cilostazol (CLZ), a selective phosphodiesterase inhibitor (PDEi), causes increase in intracellular level of cyclic adenosine monophosphate (cAMP) (9). Several investigation in different cells and tissues have indicated to inhibitory effect of CLZ on ROS and superoxide generation as well as its positive effect on hydroxyl radicals scavengering (10, 11). Also there are evidences that CLZ inhibited lipid peroxidation in brain tissue (12), and reduced oxidative stress through decrease the MDA level and improved glutathione level in blood of diabetic patients (13) 

It has demonstrated that increase in cAMP level, can reduce the oxidative stress effect on different cell (14). Cilostazol as a PDEi elevates intracellular level of cAMP (13). Also according to other studies, progesterone through its non-genomic effects increases the cAMP level (7). Since both of these compounds are able to enhance level of cAMP, the present study was performed to evaluate the protective effects of CLZ and PRO alone and in combination on H_2_O_2_-induced islet cells damage.

## Experimental

*Animals*

84 Male NMRI mice (25–30 g) (7 mice in each group) were obtained from animal house of Ahvaz Jundishapur University of Medical Science (Ahvaz, Iran) and housed in cages (22 ± 2 °C, under a standard 12 h light: 12 h dark cycle) and allowed *ad libitum *feed access. All experimental protocols were performed according to standards for animal care, established by the ethical committee of Ahvaz Jundishapur University of Medical Sciences (Ahvaz, Iran).

*Isolation of mice pancreatic islets*

Pancreatic islets were isolated from overnight-fasted male NMRI mice by Lacy and Kostianovsky modified collagenase digestion method (15). In brief, after cervical dislocation, abdomen of animals was opened. The common bile duct (CBD) was occluded at distal end close to the duodenum and 5 mL of Hank’s Balanced Salt Solution (HBSS) [all units in mmol/l: 115 NaCl, 10 NaHCO_3_, 5 KCl, 1.1 MgCl_2_, 1.2 NaH_2_PO_4_, 2.5 CaCl_2_, 25 HEPES, and 5 D-glucose, pH 7.4 as well as 1% BSA (Merck, Germany)] containing 1.4 mg/mL of collagenase P (Roche, Germany) was injected into the duct (16, 17).

After removal of the pancreas, it was placed into a 50 mL conical tube and incubated for 15 min at 37 °C water bath. Thereafter 15 mL cold Hank’s solution was added to tube to dilute collagenase concentration and stop further digestion process. For washing the collagenase from islet tissues, the tube was centrifuged for 2 min at 1200 rpm and supernatant has discarded. The washing procedure of islets, repeated again and remainders were transferred to a blackened petri dish (17). The islets were separated by handpicking under a stereomicroscope (Euromex, Holland) and were cultured in RPMI-1640 medium (Gibco, USA) which supplemented with 10% fetal calf serum, 100 U/mL penicillin, 100 U/mL streptomycin, 5 mM D-glucose was and gassed with 95% O_2_ - 5% CO_2_ atmosphere.

*Insulin secretion measurement*

Insulin secretion was evaluated in a glucose static incubation. The islets were preincubated overnight in RPMI medium. Isolated mouse islets were divided into six groups in separated well, as each group contain 7 islets: (1) control islets cultured for 48 h (18), (2) islets cultured for 48 h and then in same medium were exposed to H_2_O_2_ 500 μM for 2 h (18), (3) islets cultured for 48 h pretreated with PRO (0.5 μM) (19), and then were exposed to H_2_O_2_ 500 μM for 2 h, (4) islets cultured for 48 h pretreated with CLZ (10 μM) (20), and then were exposed to H_2_O_2 _500 μM for 2 h, (5) islets cultured for 48h pretreated with PRO (0.5 μM)+CLZ (10 μM) together and then were exposed to H_2_O_2_500 μM for 2 h, and (6) islets cultured for 48 h pretreated with Glibenclamide (GLB. 10 μM) (21), and then were exposed to H_2_O_2_ 500 μM for 2 h (All drugs purchased from Sigma, USA). Then islets in all groups were washed with HBSS and 1 mL culture medium including glucose concentration of 2.8 mM (basal concentration of glucose) or glucose concentration of 16.7 mM (stimulatory concentration of glucose) were added to them and incubated in 37 °C in this status for 1 h (18). Ultimately, the supernatant has taken and assayed by immunoradiometric assay (IRMA) method (DIA source INS-IRMA Kit, Neuve Belgium. C.N: KIP1251) for insulin concentration. The kit has detection limit of 1 μIU/mL, intra assay coefficient of variation (CV) of 2.1% and inter assay CV of 6.5%. Results were expressed as microU/islet/60 min for evaluating insulin secretion.

*Preparation of the samples for biochemical analyses*

For biochemical analysis, islets were isolated from overnight-fasted Male NMRI mice and divided to 6 groups similar to those previously mentioned in the insulin secretion measurement protocol in this study, but in this analysis each group contained 50 islets. At the end, islets were incubated 1h in medium containing 2.8 mM or 16.7 mM concentration of glucose (basal and stimulatory), then islets washed three times with ice-cold phosphate-buffered saline (PBS) and lysed through sonication (heilscher - Gmbh, Germany) for 10 s and maintained at 4 °C followed by 10 min centrifugation (18). Supernatants were used immediately for assaying SOD and CAT activities as well as MDA level as follow: 

*MDA level measurement*

Levels of malondialdehyde of islet tissues were measured according to this method (22) by monitoring the thiobarbituric acid reactive substance (TBARS) formation. Five hundred micro liter islets tissue supernatant was added to 1.5 mL trichloroacetic acid (10%). After centrifuging, supernatant (1.5 mL) was blended with 2 mL thiobarbituric acid (0.67%) and placed for 15 min in boiling water bath. After the sample has cooled, it was extracted with 2 mL *n*-butanol followed by centrifuge (at 4000× g for 15 min). The organic phase removed and the absorbance was read at 535 nm (spectrophotometer) using a blank containing all the reagents except the sample. Values were expressed as nmol/mg protein. The MDA standard curve was prepared using various dilution of 1,1,3,3-tetramethoxypropan.

*Determination of Catalase activity*

For measurement of CAT activity of islet tissues, we used the Claiborne᾽s method (23). Initially, a mixture of 50 mM potassium phosphate (pH 7.0), 19 mM H_2_O_2_, and 20 mL supernatant of homogenate pancreas islets, was prepared. The H_2_O_2_ was added to this mixture at the end, for beginning the reaction. The rate of H_2_O_2_ decomposition was assessed by measuring the absorbance changes at 240 nm for 60 s. One unit of CAT activity is defined as 1 μM of hydrogen peroxide that is consumed in 1 min. ultimately the specific activity of CAT was expressed as unit/mg protein.

*Determination of Superoxide dismutase activity*

The SOD activity in pancreatic islet tissues was estimated by applying the Suttle᾽s method (24) with the SOD kit (Randox Labs, Crumlin, UK). The principle of this method is based on SOD ability for inhibiting the reduction of nitroblue tetrazolium (NBT) and formation of red formazan which its concentration is measured by spectrophotometer at 505 nm. 50% inhibition of NBT reduction of the enzyme is defined as 1 unit SOD. The SOD activity was expressed as unit per milligram protein. 

*Protein measurement*

The concentration of protein was assayed according to Bradford method (25). Briefly, a volume of 20 μL prepared sample was added to 1 mL Bradford reagent. Then the light absorbance was measured after 5 min in 595 nm. Bovine serum albumin was used as standard.

*Statistical analysis*

Data are expressed by SPSS as mean ± SEM. One-way Analysis Of Variance (ANOVA) was used for comparison of data of different groups, followed by Tukey᾽s test. p-value < 0.05 was considered as statistically significant differences. 

## Results and Discussion

*Effects of PRO, CLZ on insulin secretion from pancreatic islets*

**Figure 1:**
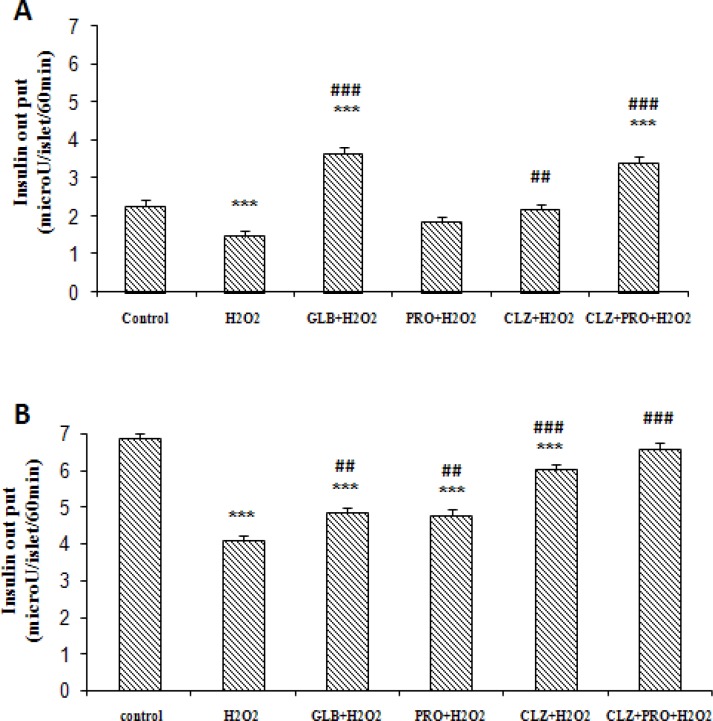
48 h Pretreatment effects of progesterone (PRO. 0.5 μM), cilostazol (CLZ. 10 μM), PRO and CLZ together, Glibenclamide (GLB. 10 μM) on insulin secretion from mice isolated pancreatic islets after 2 h exposure of islets to H_2_O_2_ (500 μM) and subsequent 1 h incubation with 2.8 mM (A) or 16.7 mM (B) glucose containing medium. (7 mice in each group). Results are expressed as mean ± SEM.

The rate of insulin secretion (microU/ islet/60 min) from isolated islets were decreased significantly in the presence of 500 μM H_2_O_2_ (1.47 ± 0.15 and 4.09 ± 0.13) in comparison with control group, (2.26 ± 0.16 and 6.84 ± 0.17) in basal (Figure 1A) and stimulatory (Figure 1B) glucose medium, respectively (P < 0.001). Also insulin secretion showed a significant increase in pretreated groups with CLZ (2.16 ± 0.15), (P < 0.01), GLB (3.65 ± 0.16) and PRO+CLZ (3.41 ± 0.14), (P < 0.001) compared to H_2_O_2_ group in basal glucose medium (Figure 1A). Furthermore a remarkable increase in insulin secretion was observed at the stimulatory glucose medium (Figure 1B) in groups which pretreated with PRO and GLB (4.83 ± 0.16 and 4.75 ± 0.17 respectively), (P < 0.01), CLZ and PRO+CLZ (5.99 ± 0.15 and 6.56 ± 0.16 respectively) (P < 0.001), compared with H_2_O_2_ alone. 

*** (P < 0.001) vs. control group. **## **(P < 0.01) and **### **(P < 0.001) vs. H_2_O_2_ group.

*Pancreatic islets MDA levels*

MDA levels (nmol/mg protein) following incubation of islets with 500 μM H_2_O_2_ increased significantly (P < 0.001) compared to control group in 16.7 mM glucose medium. Also 48 h treatment of islets with PRO+CLZ and GLB decreased (P < 0.001) MDA level as compared to H_2_O_2_ group in 2.8 mM glucose medium (Table 1). Relative to H_2_O_2_ group in 16.7 mM glucose medium, significant decrease in MDA level were observed (P < 0.001) in all pretreatment groups. The effect of PRO+CLZ was better than other groups (Table 2). 

**Table 2:** The Effects of progesterone (0.5 μM), cilostazol (10 μM), Glibenclamide (10 μM) pretreatment on the MDA level, SOD and CAT activities of mice isolated pancreatic islets following 2 h exposure of islets to H_2_O_2_ (500 μM) and subsequent 1h incubation with 16.7 mM glucose containing medium. (7 mice in each group).

**Enzyme**	**Catalas activity ** **(U/mg Protein) **	**SOD activity ** **(U/mg Protein) **	**MDA ** **(nmol/mg Protein) **
**Groups**			
Control	4/06 ± /18	37/96 ± 4/9	6/22 ± /08
H_2_O_2_	1/5 ± /12 ***	17/66 ± 3/6 ***	9/8 ± /17 ***
H_2_O_2_+ GLB	3/12 ± /12 *** ###	31/31 ± 5 * ###	7 ± /199 ###
H_2_O_2_+ PRO	2/05 ± /11 *** #	25/03 ± 3/9 ***	8/2 ± /19 *** ###
H_2_O_2_+ CLZ	2/53 ± /1 *** ###	27/69 ± 3/5 *** ##	7/8 ± /13 *** ###
H_2_O_2_+ CLZ + PRO	3/54 ± /15 * ###	36/67 ± 4/44 ###	4/6 ± /2 *** ###

H_2_O_2_: hydrogen peroxide. GLB: Glibenclamide. CLZ: cilostazol. PRO: progesterone

Results are expressed as mean ± SEM

* (P<0.05) and

*** (P<0.001) vs. control group.

# (P<0.05) and

## (P<0.01) and

### (P<0.001) vs. H_2_O_2_ group.

*Pancreatic islets CAT levels*

According to acquired results, after addition of high and basal glucose mediums to H_2_O_2_ group, CAT activity of islet tissues (unit/mg protein) decreased significantly compared to control groups (P < 0.001). Under 2.8 mM glucose, all pretreated drugs were able to increase (P < 0.001) CAT activity relative to H_2_O_2 _group (Table 1). After addition of 16.7 mM glucose medium, remarkably increase in CAT activity was observed in PRO pretreated (P < 0.05), and also other pretreated groups (P < 0.001) in comparison with H_2_O_2_ group (Table 2). 

*Pancreatic islets SOD levels*

After addition of 2.8 mM glucose (Table 1) and also 16.7 mM glucose containing mediums (Table 2), islets SOD activity (unit/mg protein) of negative control group, significantly decreased compared to control group (P < 0.001). Following the addition of basal glucose medium (Table 1), significant improvement of SOD activity was shown in PRO (P < 0.01) and other pretreatment groups (P < 0.001) compared to H_2_O_2_ group. Pretreatment with CLZ (P < 0.01), PRO+CLZ and GLB (P < 0.001), increased SOD activity in the stimulatory glucose medium (Table 2) compared to H_2_O_2_ group. 

In this study, H_2_O_2_ is used as a substitute of ROS for evaluation of ROS effects on islet cells function. This model has also been used widely in previous researches for assessing the role of ROS (26). In our investigation, exposure of mice islets to H_2_O_2_ significantly decreased insulin release from them after addition of 2.8 mM and also 16.7 mM glucose mediums. Our findings are consistent with Xiong and *et al. *report (18) which showed that H_2_O_2_ damages islet cells and subsequent decreases their insulin secretion. The present study also exhibited that pretreatment of H_2_O_2_ -induced damaged islets with PRO, increased insulin secretion. In agreement with our study, Shao and *et al*. reported that PRO stimulates insulin secretion in MIN6 β-cells (27). Also similar excitatory effect on insulin secretion has been achieved by Hollew and *et al. *after 20 h incubation of rat islets with PRO (28). However, Sorenson and *et al. *have shown the inhibitory effect of PRO on insulin secretion from neonatal rat islets (9). Although the accurate cause of this differential response to PRO is not apparent, but it may result from the different kinds of islets that have been studied. In the medium culture, neonatal islets may not be able to respond efficiently to glucose stimulation for insulin secretion and this procedure perhaps is due to immaturity of the metabolic system of neonatal pancreatic beta cells (29).

Also in our study pretreatment with CLZ remarkably increased insulin secretion as compared with H_2_O_2_ group. The present study showed that combined pretreatment of PRO and CLZ has a stronger effect on insulin secretion from islets.

Because of the vulnerability of pancreatic islets to oxidative damage, exposure of them to ROS can activate several cellular stress-sensitive pathways that have been linked to decreased insulin secretion (30). Our results in this study showed that H_2_O_2_ as a substitute of ROS, decreased insulin output of pancreatic islets by induction of oxidative stress. Administration of compounds with antioxidant properties can augment the defense capacity of islet cells to deal with oxidative stress (31). Likely pretreatment of islets with CLZ or PRO, to some extent restored secretary function of islets through potentiating of islets antioxidant defense system. On the other hand combination of CLZ and PRO represent higher antioxidant properties and so better preserve islets function.

The rate of lipid peroxidation and destruction of the cell membrane was assayed by evaluation of MDA level (32). At this study, expose to H_2_O_2_ (as an oxidative stress inducer) significantly increased the MDA level of islet cells of H2O2 group after addition of stimulatory concentration of glucose medium in comparison with the control group.

Also several studies which have investigated the toxicity effects of Interleukin1-β, tumor necrosis factor-α and interferon gamma on isolated islets of rats and human, demonstrated that this cytokine mixture induced significant increases in MDA and decreases in insulin content in islets. In addition, an antioxidant compound (lazaroid U78518E) inhibited this cytokine effects, decreased MDA level and increased insulin of islets (33, 34)

Our findings indicated that the islets MDA level in 16.7 mM glucose medium was higher compared to 2.8 mM glucose medium. It is probably because high concentration of glucose, itself leads to increased ROS production, oxidative damage, as well as cell toxicity. Subsequently this results in more lipid peroxidation and higher MDA generation (35).

Also pretreatment with PRO+CLZ reduced lipid peroxidation and MDA generation in islet cells after addition of 16.7 mM glucose medium and this may partly protect the islets against H_2_O_2_-induced damage. Pretreatment with PRO+CLZ resulted in more decrease in lipid peroxidation and thus further resistance of islets cells versus free radical damages rather than alone pretreatment of them.

Our results showed that in H_2_O_2_ group, the antioxidant enzyme activities including SOD and CAT significantly decreased relative to control groups and this pointed to the oxidative effect of H_2_O_2_ in islet cells. Markus and *et al*. have indicated to similar effect of H_2_O_2_ on insulin - producing RINm5F cells, and also cited that over expression of these antioxidant enzymes, reduced H2O2 – mediated toxicity in these cells (36). 

In another study, Lortz and *et al*. investigated the combination effect of IL-1β, TNF-α and IFN-γ on insulin-producing RINm5F cells. They showed that this compound significantly decreased cell viability. Over expression of cytoprotective enzymes such as CAT and SOD protected against toxicity of the cytokine mixture due to inactivation of generated ROS in this cells (37).

Pretreatment with PRO and CLZ elevated CAT activity as compared with H_2_O_2_ group. Also pretreatment with CLZ alone or with PRO, improved the SOD activity relative to H_2_O_2_ group after addition of 16.7 mM and 2.8 mM glucose medium. However after addition of basal or stimulatory glucose mediums in our investigation, combined of PRO and CLZ has stronger effect than single pretreatment of them on antioxidant enzymes activities. As observed in this study, the PRO+CLZ effect on lipid peroxidation as well as their antioxidant effect was more impressive than single pretreatment of them.

It has also been demonstrated that increase in intracellular cyclic nucleotides such as cAMP, could decrease reactive oxygen generation, oxidative stress and subsequent development of cellular dysfunction (14). On the other hand, as mentioned previously, PRO and CLZ probably by elevation of cyclic AMP and also activation of enzymes such as PK-A, could potentiate the cell defense systems against oxidative stress. Apparently, simultaneous pretreatment of PRO and CLZ rather than alone pretreatment of them, create a higher cAMP level, and thereby stronger antioxidant properties as well as more protective effects following oxidative damages in pancreatic islets.

The main limitation in this work is the loss of some intact islets at the end of isolation procedure due to the unavoidable prolonged time necessary for islet isolation.

In conclusion, the present study showed the protective effects of PRO and CLZ against H_2_O_2_ induced oxidative damage in pancreatic islets. Based on the results of our study, this effect might be mediated through a reduction of lipid peroxidation and elevation of antioxidant enzyme activities. Anyway, further studies seem required to determine the precise molecular mechanism (s) by which combination of these drugs, affect on pancreatic islets. 
